# CYP21A2 Gene Mutations in Congenital Adrenal Hyperplasia: Genotype−phenotype correlation in Turkish children

**DOI:** 10.4008/jcrpe.v1i3.49

**Published:** 2009-02-02

**Authors:** Firdevs Baş, Hülya Kayserili, Feyza Darendeliler, Oya Uyguner, Hülya Günöz, Memnune Yüksel Apak, Fatmahan Atalar, Rüveyde Bundak, Robert C. Wilson, Maria I. New, Bernd Wollnik, Nurçin Saka

**Affiliations:** 1 Istanbul University, Istanbul Faculty of Medicine, Department of Pediatrics, Pediatric Endocrinology Unit, Istanbul, Turkey; 2 Istanbul University, Istanbul Faculty of Medicine, Medical Genetics Department, Istanbul, Turkey; 3 Istanbul University, Institute for Experimental Medicine, DETAE, Istanbul, Turkey; 4 Mount Sinai School of Medicine, Department of Pediatrics, New York, NY, USA; 5 Center for Molecular Medicine Cologne, and Institute of Human Genetics, University of Cologne, Cologne, Germany; +90−212 532 42 33+90−212 533 13 83firdevsbas@superonline.comİstanbul Tıp Fakültesi, Çocuk Kliniği, Çapa 34390, İstanbul, Turkey

**Keywords:** 21−hydroxylase, congenital adrenal hyperplasia

## Abstract

**Background**: Congenital adrenal hyperplasia (CAH) due 21−hydroxylase deficiency (21−OHD) is a common autosomal recessive disorder. It is caused by defects in the CYP21A2 gene.

**Objective**: Our aim was to determine the frequency of common gene mutations and to evaluate genotype−phenotype correlations in Turkish 21−OHD patients.

**Methods**: Molecular analysis of the CYP21A2 gene was performed for the detection of the eight most common point mutations [p.P30L, IVS2−13C>G (IVS−2), p.I172N, exon 6 mutation cluster (p.I236N, p.V237E, p.M239K), p.V281L, p.Q318X, p.R356W, 8−bp−deletion], of large deletion and conversion by southern blotting, allele specific semi−quantitative PCR/enzyme restriction method and sequencing,  in 56 patients with 21−OHD, from 52 families.

**Results**: Disease−causing mutations were identified in 77 out of 91 alleles (84.6%) of the patients. Mutations were found in 34 of 43 alleles (79.1%) in salt wasting (SW; n=26), 32 of 36 alleles (88.8%) in simple virilizing (SV; n=24) and 11 of 12 alleles (91.6%) in non−classical (NC; n=6) form of CAH.  The most frequent mutations were IVS−2 (22.0%), large conversion (14.3%), p.I172N (9.9%) p.R356W (8.8%), and large deletion (6.6%). In the SW form, the most frequent genotypes were homozygous for  IVS−2 (11.5%) and homozygous for large conversion of the gene (11.5%). In the SV form, the most frequent genotype was homozygous for IVS−2 (20%), followed by compound heterozygous for p.I172N/8−bp del (10%). Homozygous for  p.V281L (16.7%) was most common in NC.  In most cases there was good correlation between genotype and phenotype. In the SW and NC forms, genotypes of all the patients correlated with their phenotypes.

**Conclusions**: This is the first comprehensive study on the molecular basis of CAH patients in the Turkish population. Based on these results, we propose a modified screening strategy to facilitate molecular testing of CAH patients in our population.

**Conflict of interest:**None declared.

## INTRODUCTION

Congenital adrenal hyperplasia (CAH) is a common autosomal recessive disorder that is frequently caused by 21−hydroxylase deficiency (21−OHD). Impaired 21−OH enzyme activity leads to a deficiency in adrenal cortisol and aldosterone production and to a concomitant increase in androgen secretion. CAH can be divided into classical and nonclassical forms. Clinically, classical CAH occurs as the salt wasting (SW) form with a complete lack of the 21−OH enzyme activity or as the simple virilizing (SV) form with partial impairment of 21−OH enzyme. In the SW form, cortisol and aldosterone deficiency may cause life−threatening hyponatremic dehydration and shock. In classical 21−OHD, prenatal androgen excess causes the development of external genital ambiguity in females. After birth, males and females with the classical form exhibit progressive postnatal virilization. Reduced fertility and menstrual abnormalities in untreated women and testicular adrenal rests in untreated men have been observed.(1, 2, 3, 4, 5, 6) Nonclassical 21−OHD (NC 21−OHD) results from a mild deficiency of the 21−hydroxylase enzyme with a reduced, but residual enzyme activity. Female NC 21−OHD patients do not demonstrate genital ambiguity at birth. Males and females may manifest variable signs of androgen excess at any phase of postnatal development.(7)

Analysis of CAH incidence from 6.5 million newborns screened in the general population worldwide, estimated the overall incidence of 1:13,000−1:15,000 live births for classical form of CAH.(8, 9) The prevalence for classical forms in specific populations has been reported as 1:10,000−23,000 in the United States and Europe(10), 1:21,000 in Japan(11) and 1:23,000 in New Zealand.(12) In two populations, the Yupik Eskimos (Alaska) and the people of La Reunion (France), a high frequency of the classical forms, 1:282 and 1:2141 respectively, has been reported.(9) The frequency of the NC form has been reported as 1:27 for Ashkenazi Jews, 1:53 for Hispanics, 1:333 for Italians and 1:1000 for other Caucasians.(7)

To date, over 100 mutations have been described in the human CYP21A2 gene which cause CAH. There are an increasing number of reports concerning the genetics of 21−OHD being published from various countries worldwide.(1, 13, 14) Approximately 95% of all disease−causing mutations in CYP21A2 gene are large deletions, large conversions or one of eight point mutations [p.P30L, IVS2−13 C>G in intron 2 splice site (IVS−2), 8bp deletion in exon 3, p.I172L, Exon 6 cluster (p.I236N, p.V237E, p.M239K), p.V281L, p.Q318X, p.R356W]. These common point mutations are due to unequal crossing over between the pseudogene, CYP21A1P and the active gene, CYP21A2.(13, 14) Large deletions or large conversions are typically associated with classical SW, whereas the IVS−2 may be associated with either the salt−wasting or simple virilizing forms. Specific missense mutations associated with other forms of 21− OHD include the simple virilizing Exon 4 I172N mutation and the non−classical mutations, p.V281L, p.P30L, p.R339H and p.P453S.(1, 3, 5, 15) Previously, several large genetic screenings have been performed with CAH families where the genotype−phenotype correlation was assessed with 80−90% accuracy.(16, 17, 18, 19, 20, 21, 22)

We present here the screening results of the most common CYP21A2 gene mutations found in our Turkish patients with CAH and an analysis of their genotype in relation to phenotype.

## SUBJECTS AND METHODS

**Subjects**

Fifty−two Turkish families with children with 21−OHD from different areas in Turkey participated in our study. Four families had two affected siblings. Therefore, there were a total of 56 affected patients studied. Fifteen of the 52 families studied demonstrated consanguinity. The number of alleles was considered as 1 in 13 of the consanguineous families. Heterozygous compound mutations were found in 2 of the families. The number of alleles was 2 in one of these families, while only one mutant allele was found in the second family. Therefore, the number of alleles were 91 in total. There was no phenotypic variation in the four families with affected siblings.

Blood samples were collected from the affected and unaffected individuals of each family. Informed consent was obtained in all cases.

**Phenotypic analysis**

Clinical and hormonal evaluations were used to categorize the patients with the SW, SV, or NC forms of CAH. It was found that our study population consisted of 26 patients diagnosed as having the SW form, 24 as having the SV form, and 6 as having the NC form of CAH. The criteria used to diagnose a SW form was either a SW crisis in the newborn period or elevated plasma renin activity (PRA) levels, hyponatremia and hyperkalemia. Phenotypic features of the SW and SV forms included growth failure in the first months of life, very early

pubarche, advanced bone age, acceleration of growth, and ambiguous genitalia in the affected female. The NC form presented with normal external genitalia, hirsutism or mild clitoromegaly in girls and, in both sexes, by precocious pubarche.

External genitalia was graded according to Prader staging (23) in all females. ACTH stimulation testing was done in some of the patients to confirm their diagnoses. After diagnosis was confirmed, hydrocortisone and fludrocortisone replacement therapies were started for SW patients and only hydrocortisone for those affected with the SV and NC forms of CAH.. Basic clinical and laboratory characteristics of patients with different forms of CAH are given in Table 1. Some patients were re−evaluated to confirm their diagnoses.

**Hormonal investigations**

Serum adrenal precursors (17 hydroxyprogesterone [17−OHP], testosterone, androstenedione, dehydroepiandrostenedionesulphate) and PRA were measured by RIA according to the procedure manual (Diagnostic Systems Laboratories, Inc, Texas, USA) at diagnosis and at follow−up.

**Genetic analysis**

DNA was extracted from peripheral blood using a commercially available kit (DNA Isolation Kit for Mammalian Blood, Roche, Istanbul, Turkey). Genetic analysis of 56 affected individuals was performed. Seven analyses were completed at the Mount Sinai School of Medicine in New York and the other 49 were completed at the Istanbul Faculty of Medicine in Istanbul. Allele−specific PCR was used to screen the patients and families for the 8 most common mutations [p.P30L in exon 1, IVS2−13C>G in intron 2, 8−bp deletion in exon 3, p.I172N in exon 4, cluster mutation in exon 6 (p.I236N, p.V237E, p.M239K), p.V281L in exon 7, p.Q318X and p.R356W in exon 8]. Southern blot analysis or semi quantitative PCR/ enzyme digestion methods were performed to detect the large deletions and conversions by the methods reported previously by Tukel et al.(24) Sequence analysis was performed on patients with only one affected allele; the p.R339H and p.P453S mutations were also detected by sequence analysis.

**Statistical analysis**

An SPSS−10 program was used for statistical analyses. Comparison between the means was done by nonparametric tests. The results were expressed as an arithmetic mean±SD. The relations between variables were analyzed by Spearman’s correlation test. The distribution of the mutations among the three clinical types were compared using chi−square tests, p20.05 was considered statistically significant.

**Table 1:**
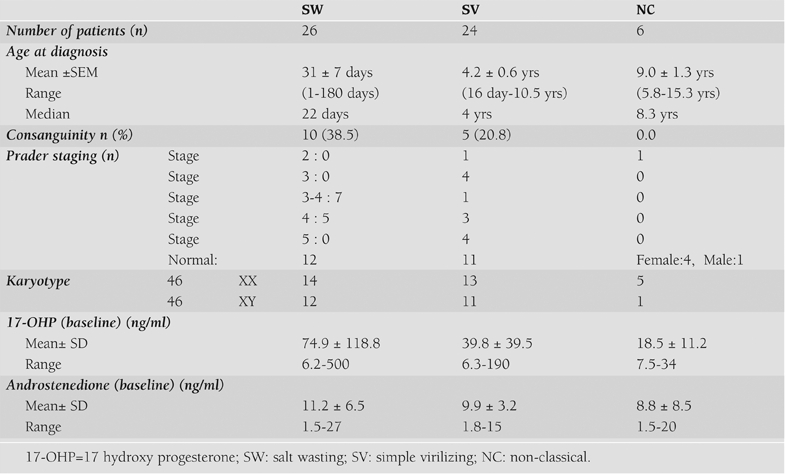
Clinical and laboratory findings of the patients with different forms of CAH

## RESULTS

Clinical and laboratory findings of these Turkish patients with CAH who were screened for genetic mutations are summarized in Table 1. Age at diagnosis of the SW patients was lower than the SV and NC patients (p<0.001). There was no significant difference in the Prader stages of the SW and the SV patients (p=0.1).

Frequency of consanguinity was 32.0% in all patients with classical CAH: 38.5% in the SW population, and 20.8% in the SV population.

17−OHP and androstenedione levels were found to be higher in those with SW than in those with SV, but the difference was not significant (p=0.743 and p=0.545, respectively). At the time of diagnosis, there were correlations between age and androstenedione levels (r=0.584, p=0.009), 17−OHP levels and birth weight (r=0.540, p=0.038), androstenedione and testosterone (r=0.892, p=0.003) in the SV patients. There were no such correlations in the SW patients.

The disease−causing mutations were identified in 77 of 91 unrelated alleles (84.6%) for the three forms of CAH: 34 of 43 alleles (79.1%) in SW, 32 of 36 alleles (88.8%) in SV and 11 of 12 alleles (91.6%) in NC. Mutations of all three forms of CAH are summarized in Tables 2 and 3, and the mutation frequencies of affected alleles in 52 unrelated patients are shown in Table 3. Among the 91 unrelated alleles, distribution of the most frequent mutations were IVS−2, large conversion of the CYP21 gene, p.I172N, p.R356W, and large deletion of the CYP21 gene, as seen in Table 3.

In the SW patients, the most frequent mutation observed was IVS−2, followed by large conversion of the gene, p.R356W, large gene deletion, and p.Q318X. The most frequent genotypes in this patient population were homozygous for IVS−2 and homozygous for large conversion of the gene. One of the SW patients had a complex allele p.[[R356W]+[V281L](+)I172N]. His mother was a carrier of the p.R356W and his father was a carrier of the V281L mutation, and I172N had occurred de novo.

In the SV patients, the most frequent mutation observed was IVS−2, followed by p.I172N, large conversion of the gene and p.R356W as seen in Table 3. The most frequent genotype was homozygous for IVS−2, followed by compound heterozygous for p.I172N/8−bp del. The p.I172N, cluster exon 6 (p.I236N, p.V237E, p.M239K), p.V281L, p.P453S, and p.R339H mutations were present among the SV patients, but absent in patients with the SW form. The p.Q318X mutation which was one of the most frequent mutations in the SW patients was not found in the SV or NC patient population. The IVS2− mutation showed a similar prevalence in both the SW and SV forms as seen in Table 3.

In the NC population, the most frequent mutation observed was p.V281L. Other mutations were IVS−2, del8bp, large gene deletion, p.I172N , p.R453S and p.R339H as seen in Table 3. In the NC form, the most frequent genotype was homozygous for p.V281L (16.7%). There were 4 compound heterozygous mutations in the NC cases: gene deletion/p.R339H, IVS−2/p.P453S, p.I172N/p.V281L, and p.V281L/8−bpdel as seen in Table 2.

In patients with the classical form of CAH, the frequency of compound heterozygous mutations was found to be 34.8% (16/46): 26.9% (7/26) in the SW form, and 45% (9/20) in the SV form. One of the SW patients showed a compound heterozygous mutation (IVS−2/large gene deletion), despite his parents being consanguineous. The frequency of homozygous mutations was found to be 41.3% (19/46) in all classical CAH patients: 42.3% (11/26) in the SW form, and 40.0% (8/20) in the SV form. The frequency of detecting only one mutation in one allele was observed to be 19.6% (9/46) in all classical CAH patients: 26.9% (7/26) in the SW form, 10.0% (2/20) in the SV form. For the NC population, the frequency of detecting only one mutation in one allele was 16.6% (1/6). There were no mutations found in both alleles in 4.3% (2/46) of all classical CAH patients: 3.8% (1/26) in the SW form, and 5% (1/20) in the SV form (Table 2).

In most 21−OHD cases there is good correlation between genotype and phenotype. In the SW and NC forms, genotypes of all participating patients correlated with their phenotypes. However, in the SV form, 5 out of 24 patients had genotypes that would predict the NC form. These 5 patients had a severe mutation on 1 allele and a mild mutation on the other (Table 2, patients 28 a, 28 b, 30, 37, 45). In addition, two sibs with the SV form had a severe mutation on both alleles (Table 2, patients 40 a and 40 b) that would predict the SW form.

**Table 1:**
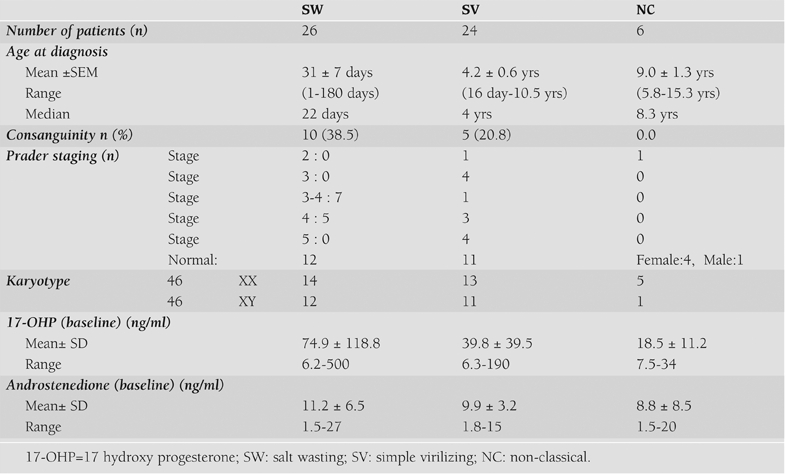
Clinical and laboratory findings of the patients with different forms of CAH

**Tables 2:**
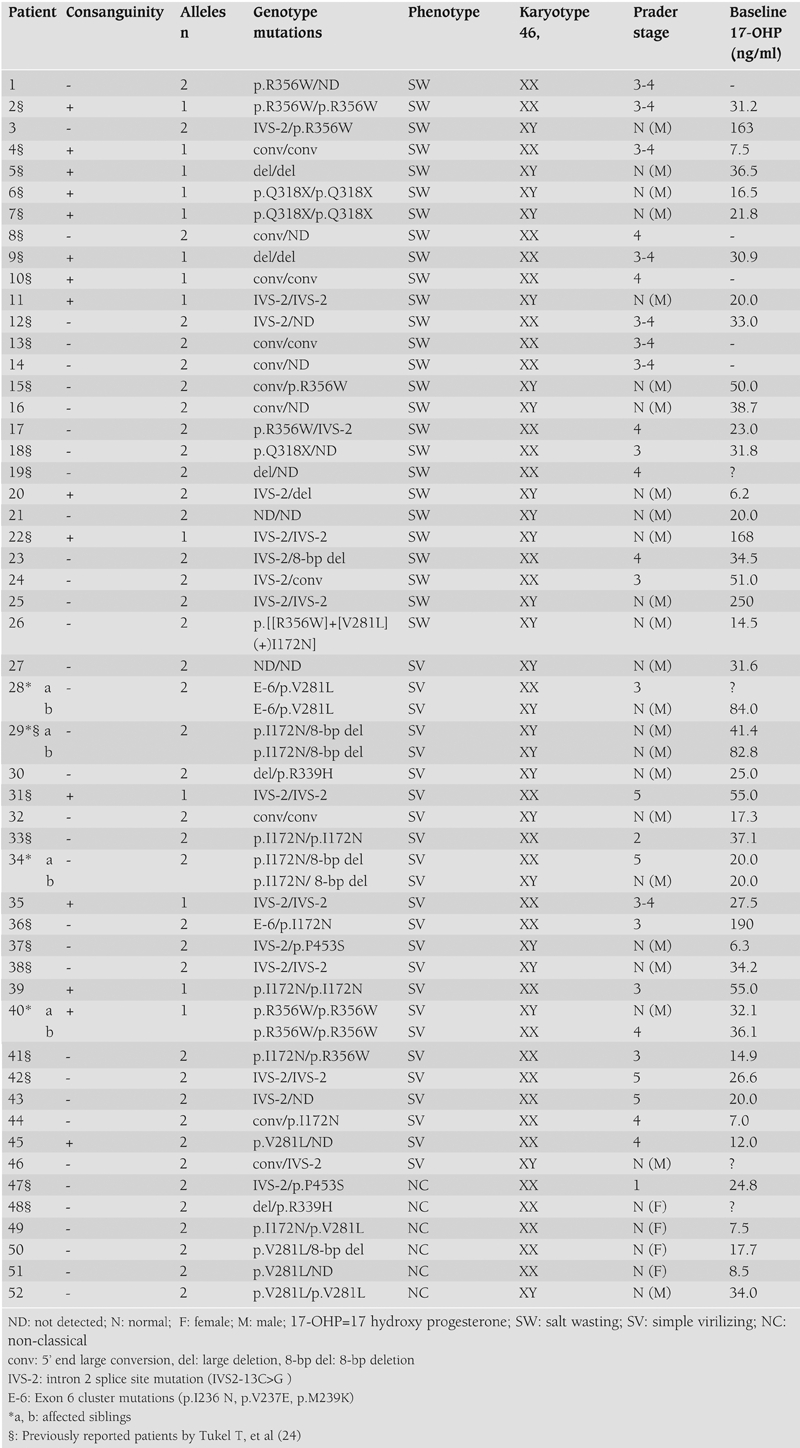
Genotype and phenotype of all patients with CAH

**3:**
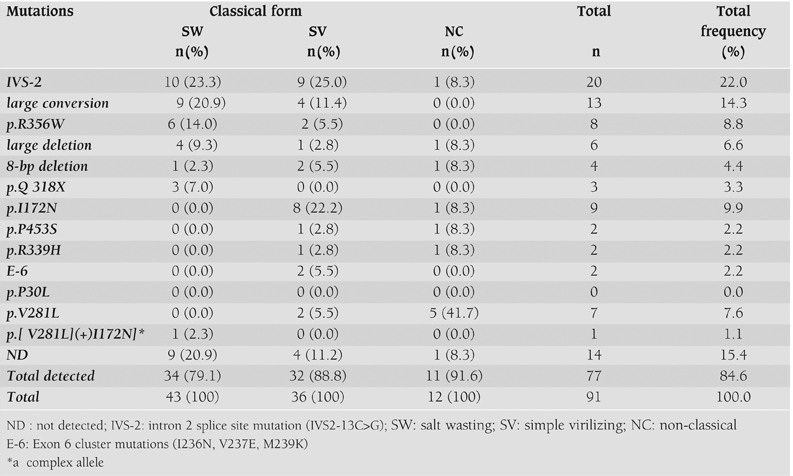
Distribution of mutations obtained in unrelated Turkish patients with CAH due to 21−OHD

## DISCUSSION

We have identified the disease−causing mutations on 77 of 91 (84.6%) alleles using the described screening methods for the most common mutations. The mutations were found on 34 of 43 alleles (79.1%) in SW, 32 of 36 alleles (88.8%) in SV and 11 of 12 alleles (91.6%) in NC. In our study, the frequency of undetectable mutations was 15.4%. This rate varied between 0 and 26.7% in other studies.(18, 20, 22, 25, 26, 27, 28, 29, 30, 31, 32, 33, 34, 35, 36, 37, 38, 39, 40, 41) The incidence of CYP21A2 gene mutations in 21−OHD has been extensively studied. Results of CYP21A2 gene mutations in different populations are summarized in Table 4.

The present study shows that the frequencies of the 8 most common mutations are similar to results obtained from Western European and American populations (Table 4). (17, 19, 20, 25, 29, 31) In our study, the frequency of p.Q318X was slightly lower as compared to the Italian population.(29) The frequency of p.V281L was higher those reported for Netherlands (31) and American populations.(25) The frequency of 8−bp del was found to be lower than the American figures (Table 4). (25) Additionally, some differences were observed in the distribution of these common mutations in Turkish patients when compared to Eastern European, Latin, Asian, Iranian, and Tunisian populations. The frequency of the IVS−2 mutation in classical CAH patients was high in Romania (43.9%) (33) and Hungary (41.3%) (42), in comparison to 24.0 % in Turkish patients with classical CAH. The most frequent mutation in the Tunisian CAH population was found to be p.Q318X (35.3%), in contrast to 0.5−13.8% described in another report.(39) In our study, the p.Q318X mutation was only found in those patients with the SW form. Also, we found that the mutation frequency of p.Q318X in the Turkish patients (3.3%) to be lower than in the Tunisian population.(39)

The frequencies of p.R356W and 8−bp del mutations were found to be 10.1% and 3.8%, respectively, in our Turkish patients with classical CAH. This differed from the figures for Iranian patients with classical CAH who demonstrated no p.R356W mutations as well as a higher frequency of the 8−bp del (10%) mutation.(40) Unlike some populations, the p.P30L mutation was not detected in Turkish patients with classical or non classical CAH.

The distribution of mutations in a newly reported study from Turkey revealed mutations in 78% patients with slight differences in the frequency of some mutations as summarized in Table 4. There were patients from different areas of Turkey except for the Marmara region in that study, (41) whereas our study included patients from all parts of Turkey.

Recently, important variations in allelic mutation frequencies were demonstrated in different ethnic groups in a large study by Wilson et al.(16) A high prevalence of the following mutations were found in the respective ethnic populations: The V281L mutation in Ashkenazi Jews, an IVS−2 mutation in Iranians and Yupik Eskimos of Western Alaska, a large gene deletion in Anglo−Saxons, a p.R356W mutation in Croatians; and a p.Q318X mutation in East Indians.(16)

There is a wide spectrum of severity of the disease with a good correlation between genotype and clinical phenotype in 21− OHD, with few exceptions. In Caucasian populations, deletions and gene conversions are associated with the SW form, whereas the IVS−2 mutation may be associated either with the SW or SV form. V281L mutation is found to be common in NC CAH.(7, 17, 18, 19, 20, 22, 14, 26, 29, 31)

In our study most cases demonstrated good correlations between genotypes and phenotypes. In the SW and NC forms, genotypes of all the patients when detected correlated with their phenotypes. However, in the SV form non−concordance of genotype/phenotype was found in 5 out of 24 patients. These patients’ genotypes (p.V281L or p.R399H or p.P453S on one allele) predicted the NC form. In addition, two sibs diagnosed with the SV form carried the homozygous p.R356W mutations which predict the SW form. In some studies, phenotypic variation has been detected. However, the phenotypic variations do not always correlate with allelic variation. For instance, patients who were homozygous for IVS−2 mutation were found to have either the SW or SV phenotype.(25, 43, 44, 45) The I172N mutation is usually associated with the SV form,(17, 18, 19) although patients with the SW form have been reported to have the p.I172N/deletion genotype.(17, 25) Phenotypic variance may be present in siblings, which suggests a role for modifiers of 21−OHD transcription, translation, and action.(46)

Wilson et al (16) observed a non concordant genotype/phenotype correlation when at least one IVS−2 mutation was present. It was suggested that the genotype/phenotype non−correlation in patients with the IVS−2 mutation might result from the variable splicing of this mutation. The variable expression could be due to variation in RNA splicing factors.(16)

In our study, a severe mutation (large gene deletion or IVS−2) was found on one allele in two compound heterozygote individuals described as having non−classical CAH. These patients are at 25% risk for having a child with classical 21−OHD if his/her partner is also a carrier of a classical mutation. Prenatal treatment and diagnosis should be offered if the partner carries a classical mutation.

The variability in the phenotypic expression of NC 21−OHD has been determined in previous studies. Compound heterozygosity with one mild and one severe mutation can lead to more severe clinical findings than carrying two mild mutations. The existence of a subgroup with compound heterozygosity for one mild and one severe mutation may have important implications for future genetic and prenatal counseling.(14, 47) One of our patients whose parents were consanguineous demonstrated compound heterozygosity. This is unusual since the offspring of consanguineous parents usually have homozygous mutations.

The compound heterozygosity in our study (34.8%) was highly similar to Tunisian (39) and German (20) patients with 21−OHD (17.6% and 72,25%, respectively), despite the high frequency of consanguinity. However, compound heterozygosity in another study from Turkey (41) was found to be lower (7%) than in our study in the presence of higher rate of consanguinity.

Some alleles can carry more than one mutation.(20, 31, 32, 33, 38, 39) In our study, one of the SW patients had a complex allele comprised of p.[[R356W]+[V281L]+[I172N]]. His mother was a heterozygous carrier for the p.R356W and his father was a heterozygous carrier for the p.V281L mutation. Neither parent had the p.I172N mutation. This mutation could have occurred as a de novo mutation originating in only one germ cell or due to gonadal mosaicism.

In 10 of our patients (6 SW, 2 SV and 1 NC), one allele was found to be affected while the other allele did not have any mutation. Wilson et al25 also reported a family in which only one mutant allele was found. CYP21A2 gene sequencing did not show any other genetic alterations, although other regions were tested for mutations (e.g. promoter regions, intronic region) on the second allele. Krone et al (20) also reported two patients in which only one affected allele was detected, although clinical and hormonal findings of these patients were compatible with 21−OHD. Other studies have detected mutations in the CYP21 promoter, (48) and transcriptional regulatory regions.(49)

Recently, Escobar−Morreale et al (50) suggested that the cutoff value for basal 17 OHP for the detection of NC CAH should be 1.7 ng/ml instead of the currently recommended 2 ng/ml as the upper limit of the normal ranges in women. However, basal 17 OHP levels in our patients with NC 21−OHD were found higher than the recommended cutoff levels. Molecular diagnosis could also be useful to resolve the problems of false negative or positive results which might occur during biochemical diagnosis in the newborn. Identification of the genotype could be a guide in planning a strategy for prenatal treatment and for newborn screening programmes. The relationship between genotype and phenotype may also be of value in evaluating response to treatment.(51)

In conclusion, this study presents the results of the first genotype−phenotype molecular study of 21−OHD in Turkish patients, the initial findings of which were reported previously.(24) The frequencies of common mutations were similar to Western European and American populations. With the exception of a few cases, a genotype−phenotype correlation has been observed in this group of patients. Based on these finding, screening policies will be instituted for the Turkish population.

We believe these data will be useful in giving enhanced patient care, in offering genetic counseling, and also in prenatal diagnosis and, treatment. They may also prove to be useful in the detectipn of carriers.Furthermore, the diagnosis of CAH will be made more accurately when based on the results of genetic testing.

**Table 4:**
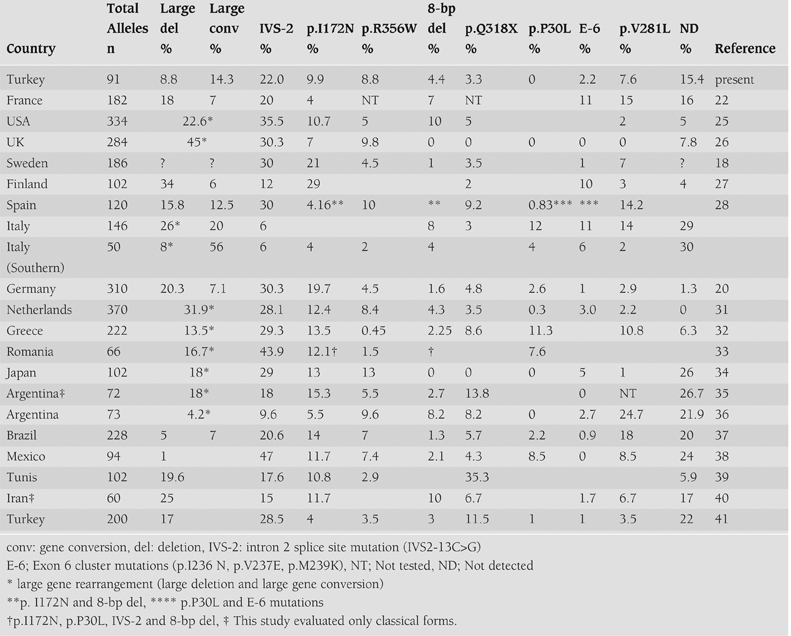
Distribution of mutations in unrelated Turkish patients affected by CAH, 21−OHD, in comparison with other populations

## ACKNOWLEDGEMENT

Molecular studies on 21−OHD have been initiated at the Medical Genetic Division of the Department of Pediatrics, Istanbul Faculty of Medicine, Turkey, by Turgut Tukel as his PhD thesis. He continued his studies in the Medical Genetics Department of Mount Sinai School of Medicine after 2001. Turgut Tukel passed away in NY in May 2006. This study was supported in part by NICHD Award No.HD00072 and NIH Award No. RR19484.
